# A product of independent beta probabilities dose escalation design for dual-agent phase I trials

**DOI:** 10.1002/sim.6434

**Published:** 2015-01-29

**Authors:** Adrian P Mander, Michael J Sweeting

**Affiliations:** a MRC Biostatistics Unit Hub for Trials Methodology Research, Institute of Public Health, University Forvie SiteCambridge, CB2 0SR, U.K.; b Cardiovascular Epidemiology Unit, Department of Public Health and Primary Care, University of CambridgeCambridge, CB1 8RN, U.K.

**Keywords:** adaptive design, dual-agent trial, nonparametric, phase I clinical trial, dose escalation

## Abstract

Dual-agent trials are now increasingly common in oncology research, and many proposed dose-escalation designs are available in the statistical literature. Despite this, the translation from statistical design to practical application is slow, as has been highlighted in single-agent phase I trials, where a 3 + 3 rule-based design is often still used. To expedite this process, new dose-escalation designs need to be not only scientifically beneficial but also easy to understand and implement by clinicians. In this paper, we propose a curve-free (nonparametric) design for a dual-agent trial in which the model parameters are the probabilities of toxicity at each of the dose combinations. We show that it is relatively trivial for a clinician's prior beliefs or historical information to be incorporated in the model and updating is fast and computationally simple through the use of conjugate Bayesian inference. Monotonicity is ensured by considering only a set of monotonic contours for the distribution of the maximum tolerated contour, which defines the dose-escalation decision process. Varied experimentation around the contour is achievable, and multiple dose combinations can be recommended to take forward to phase II. Code for R, Stata and Excel are available for implementation. © 2015 The Authors. *Statistics in Medicine* Published by John Wiley & Sons Ltd.

## 1. Introduction

Dual-agent phase I trials are becoming increasingly commonplace in the drug development process as more drugs are used in combination. Combination therapies may offer potentially synergistic benefits, and the agents may target different modes of action, or using one drug may reduce the severity of side effects from the second agent. Nevertheless, a common goal in oncology trials is to maximise the dosage of both agents subject to toxicity constraints and hence obtain one or more maximum tolerated dose (MTD) combinations to take forward for further experimentation.

Model-based adaptive designs for phase I dose-escalation studies are becoming more widespread but are still not regularly used in practice. This may be especially true in dual-agent trials where the complexity and interpretability of the underlying dose-toxicity model coupled with lack of user-friendly software and computational demands required to assess operating characteristics create barriers for implementation. Many proposed dual-agent dose-toxicity models take a parametric form with escalation decisions based on the presence or absence of a dose-limiting toxicity (DLT). Various parametric forms have been proposed for dual-agent dose-toxicity models. These include a six-parameter logistic-type model [1], copula models [2,3], simple dual-agent extensions of continual reassessment method (CRM) models [4,5] and reducing the ordering of the two drugs into a single dimension and applying the CRM [6]. Braun and Wang [7] placed beta distributions on each discrete dose combination and linked the parameters of the beta distributions using two log-linear models for the number of events and nonevents, respectively.

Gasparini [8] proposes a set of ‘admissibility conditions’ that such models should satisfy, including monotonicity (i.e. the risk of DLT strictly increases with dose) and conditions whereby the functional form reduces to the marginal risks when one drug is omitted. Parametric forms should also be flexible enough to allow both synergy and antagonism. However, even if these admissibility conditions are satisfied, the models may be too complex for applied use. In particular, there is often prior information on the toxicity of each agent, especially from monotherapy trials, and incorporating this through the model parameters in a coherent way can often be challenging if the model is complex. Additionally, model misspecification, either in the model or the priors, can lead to unsafe dose escalations [9].

Wang and Ivanova [4] argue that the choice of the model is arbitrary as long as it provides adequate local fit. However, a dual-agent trial may aim to target more than one MTD, and the model must therefore be flexible enough to encompass this. In this paper, we revisit the idea of a maximum tolerated contour (MTC) [10], which is formed by dose combinations that all have a probability of DLT equal to a prespecified target toxicity level (TTL). A nonparametric modelling approach is therefore appealing because it requires no assumptions to be made regarding the shape of the MTC.

Nonparametric or ‘curve-free’ models have been proposed in the past, both in single-agent [11] and dual-agent trials [12]. These models only assume monotonicity, a constraint imposed through the prior specification, although even this constraint can make the models computationally difficult. The aim of this paper is to extend these ideas but to allow prior distributions for each dose combination to be unconstrained thereby allowing a very simple specification of prior risk for any dose combination. Our proposed model is considered a ‘working model’ because the DLT risks themselves do not necessarily satisfy monotonicity, although all dose-escalation decisions are based on the monotonicity assumption. The approach is similar to the toxicity probability interval (TPI) method [13,14], where an independent beta/binomial Bayesian model is used for each dose. The difference lies in the decision algorithm used for escalation. The TPI method bases escalation decisions on probability intervals from the posterior distribution of the currently treated dose. The TPI method allows the pre-specification of all possible future decisions given the emerging data, allowing clinicians to use spreadsheets to look up next escalation steps as data accrue during the study. However, in a dual-agent setting, there are potentially too many possible escalation choices in order for this approach to be practical. Our proposed approach uses posterior probabilities from all proposed dose combinations to inform escalation, thus allowing the direction of dose escalation within a two-dimensional dose-toxicity space to be determined. A product of independent beta probabilities escalation (PIPE) strategy is proposed and described in detail in Section 2. The approach is computationally very fast, being based only on conjugate Bayesian inference. In Section 3, we show that the design has favourable operating characteristics that are comparable with other parametric designs proposed in the literature [1–3]. To aid implementation, we provide relevant software in R, Stata and implement the contour estimation within an Excel spreadsheet.

## 2. The product of independent beta probabilities escalation design for dual-agent dose escalation

The aim is to design a dual-agent dose-escalation phase I trial targeting a MTD contour; the risk of toxicity for dose combinations on this contour is a prespecified TTL, *θ*. Label this contour the *MTC*_*θ*_. We refer to the *MTC*_*θ*_ as any line partitioning the dose combination space into toxicity risks above *θ* or less than or equal to *θ*. For a discrete set of dose combinations, there are a finite number of locations that a contour can partition the space.

The objectives of the design are fundamentally different to many other phase I trial designs, because the aim is to target, experiment and recommend multiple potential doses along the *MTC*_*θ*_ rather than focusing on a single candidate, for example, as discussed in the parametric designs presented in [5].

Let 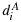 represent the dose of drug A at level *i* and 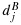 represent the dose of a second drug B at level *j*, where doses increase with *i* and *j* and *i* = 1,…,*I*, *j* = 1,…,*J*. Let *d*_*ij*_ represent the dose combination 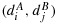. We assume that all combinations are available for experimentation in the planned phase 1 trial, with the probability of a DLT at dose combination *d*_*ij*_ represented by *π*_*ij*_.

To begin, we place independent beta prior distributions on each *π*_*ij*_ such that


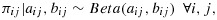


for some fixed hyperparameters *a*_*ij*_ and *b*_*ij*_. Patients are recruited into the trial sequentially in cohorts of a specified size with each cohort assigned a dose combination chosen by the dual-agent design. Suppose after the first *m* cohorts we have observed 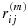 DLTs from 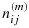 patients for dose combinations *d*_*ij*_; the data up to the end of the *m*th cohort are defined by 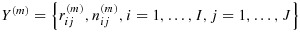. Then, assuming patients are independent with a common dose-specific DLT probability, the likelihood is binomial. Because of conjugacy and prior independence of the *π*_*ij*_, the posterior distribution of *π*_*ij*_ is also a beta distribution


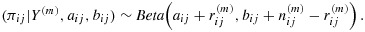


Under the assumption of monotonicity, toxicity risk increases with increasing dose, that is, *π*_*ij*_<*π*_(*i* + 1)*j*_*i* = 1,…,*I* − 1,∀*j* and *π*_*ij*_<*π*_*i*(*j* + 1)_∀*i*,*j* = 1,…,*J* − 1. Therefore, we require the estimated *MTC*_*θ*_ to be a contour that does not contradict this assumption, and hence, for escalation decisions, we need only consider contours that satisfy monotonicity (such contours will be called the monotonic contours). Nevertheless, our assumption of independent beta priors on each *π*_*ij*_ does not place any monotonicity constraints directly on the toxicity risks. In this sense, our model is considered a ‘working model’ because the DLT probabilities, *π*_*ij*_, are not constrained to be monotonically increasing with dose.

**Figure 1:**
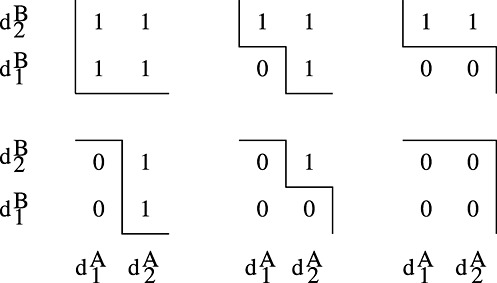
The six monotonic *MTC*_*θ*_ for two drugs, each with two experimental dose levels.

To illustrate the PIPE design, consider the situation where each drug has only two dose levels of experimentation. There are only six possible monotonic contour choices for the *MTC*_*θ*_, as shown in Figure 1. Each contour is represented by a binary matrix indicating whether doses are above the contour, that is, doses with toxicity greater than the target and assigned a value of 1 in the binary matrix, or below the contour (assigned a value of 0 in the binary matrix). In the upper-left plot of Figure 1, all the dose combinations have toxicity risk above *θ*, whilst in the lower right plot, all dose combinations have toxicity risk at or below *θ*. We will define the set of all monotonic contours as 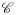. In general, for a *I* × *J* matrix, the size of 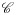 is 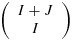 (Supporting Information) and is much smaller than the size of the set of all possible contours 2^*I* × *J*^. Let the binary matrices that are members of the set 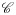 be *C*_*s*_, where 
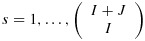
.

To find the most likely contour for the *MTC*_*θ*_, consider the posterior probability, after *m* cohorts, that the toxicity risk is less than or equal to *θ*, for any dose combination (*i*,*j*):


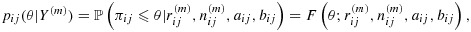


where *F*() is the cumulative distribution function of a beta distribution. Hence, the upper-left contour in Figure 1, where the risk of DLT for each dose combination is above *θ*, has the following posterior probability:


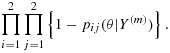


A general formula for the probability that the *MTC*_*θ*_ is the contour defined by matrix *C*_*s*_, after *m* cohorts have been recruited, can be written as


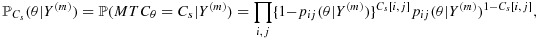
(1)

where [*i*,*j*] refers to the element of a matrix in row *i*, column *j*. Equation (1) can be derived from a decision theoretic rationale as the expected joint posterior gain over the independent dose combinations, where the gain function for combination (*i*,*j*) and contour *C*_*s*_ is given by


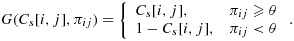


Thus, the expected posterior gain for deciding that dose combination (*i*,*j*) is unacceptable (i.e. *C*_*s*_[*i*,*j*] = 1) is 1 − *p*_*ij*_(*θ*|*Y*^(*m*)^), and for deciding that the combination is acceptable (i.e. *C*_*s*_[*i*,*j*] = 0) is *p*_*ij*_(*θ*|*Y*^(*m*)^). As all dose combinations are independent, the joint gain function is just the product of the dose-specific gain functions, and the expected joint gain function is therefore Equation (1).

Equation (1) is a product of independent beta tail probabilities, giving the PIPE design its name. The underlying rationale behind the PIPE method is that dose-escalation decisions are based on the most likely choice of *MTC*_*θ*_. The most likely contour for *MTC*_*θ*_ after *m* cohorts have been recruited is the contour that maximises the expected posterior joint gain


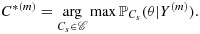
(2)

Although it is possible to have multiple most likely contours, priors can be selected to avoid this. One key advantage of using the PIPE design is that it is computationally fast, because it does not require MCMC methods to calculate the posterior probabilities. Furthermore, the design is ‘curve-free’ because it assumes no underlying dose-toxicity model.

### 2.1. Dose selection strategies

#### 2.1.1. Admissible doses

**Figure 2:**
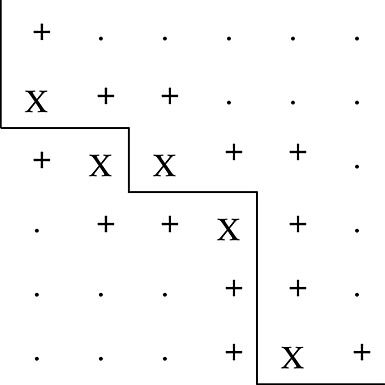
The set of admissible doses that are *closest and adjacent* (X) and *adjacent but not closest* (+) to C^*(*m*)^.

We wish to use C^*(*m*)^ to guide dose escalation and to choose from a set of dose combinations that are close to C^*(*m*)^. Figure 2 shows an example for two drugs with six levels each; the solid line is C^*(*m*)^. The dose combinations indicated by *X* are the *closest* doses to C^*(*m*)^ with respect to toxicity risk. Alternatively, we may wish to pick from a wider range of doses that lie *adjacent* to C^*(*m*)^; these dose combinations are those indicated by either X or + in Figure 2. Let Ω^(*m*)^ be the set of admissible doses, based either on the closest or adjacent dose strategy. Note that under the assumption of monotonicity, the closest dose combinations dominate all the adjacent dose combinations.

#### 2.1.2. Selecting amongst the admissible doses

One of the admissible dose combinations should be chosen as the dose for the next cohort. One way to do this is to select the next dose combination to be the admissible dose with the smallest current sample size, where sample size here is defined as both the prior and trial sample size combined, that is, 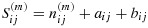. That is, we select dose *d*_*ij*_ where


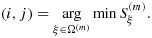
(3)

If multiple doses are returned by this function, then the dose combination administered is selected randomly from this set.

Another possible dose selection rule is based on a weighted randomisation, where the selection of the admissible doses is weighted by the inverse of their sample size, that is,


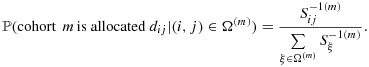
(4)

The rationale for using the inverse of the sample size to select amongst admissible doses is that it allows varied experimentation around the estimated *MTC*_*θ*_. Varied experimentation may allow the contour to be estimated more consistently [15].

It is possible to modify the rules described previously so that dose selection follows a *coherent* strategy [16], in that an observed DLT will likely lead to a de-escalation of one or both drugs, and no DLT will likely lead to an escalation. This can be achieved by further restricting the admissible dose set to closest or adjacent doses above/below C^*(*m*)^ depending on whether the current dose combination is below/above C^*(*m*)^. This strategy is generally coherent because C^*(*m*)^ adapts from C^*(*m* − 1)^ to lie above or below the current dose depending on its observed outcome.

### 2.2. Prior distributions

In most situations, priors are chosen to be minimally informative so that the likelihood contributions will quickly dominate the prior once patients have been recruited to each dose combination. The strength of the prior at each dose combination is determined by *a*_*ij*_+*b*_*ij*_; this is equivalent to the number of patients observed at dose (*i*,*j*) before the trial begins. The parameters *a*_*ij*_/(*a*_*ij*_+*b*_*ij*_) and *b*_*ij*_/(*a*_*ij*_+*b*_*ij*_) represent the proportion of DLTs and non-DLTs expected at dose combination (*i*,*j*), respectively.

To translate a toxicity contour, such as the *MTC*_*θ*_, to the prior parameters of the beta distribution is relatively trivial. At the beginning of the trial, the trialist should be asked to record which dose combinations they believe lie on or are closest to the *MTC*_*θ*_ contour. These dose combinations should then have their prior median risk set to *θ*. This will ensure that at the beginning of the trial, the *MTC*_*θ*_ contour will be adjacent to these doses. The trialist may also wish to set (prior median) toxicity risks for the lowest and highest dose combinations and any other dose combinations they have prior information on (e.g. single-agent doses). The remaining grid of prior median probabilities can then be filled in using, for example, interpolation. The final specification required is the prior strength (sample size) associated with each dose combination. In the designs that follow, we consider two possible strengths of prior: (i) a strong prior where *a*_*ij*_+*b*_*ij*_=1,∀*i*,*j* and (ii) a weak prior where 
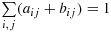
. Alternatively, the prior strength need not be equal across the dose combinations and in particular may be higher at the margins, because of incorporation of historical data from single-agent trials. The dose selection procedure described in Section 2.1.2 would then automatically explore less known areas of the dose combination space.

To calculate the hyperparameters of a beta distribution from the prior strength *s* (*s* = 1 for the strong prior and *s* = 1/(*IJ*) for the weak prior) and prior median *med*, we use an optimisation algorithm to solve *F*(*med*;*a*,*s* − *a*) = 0.5 for the parameter *a*; this optimisation algorithm is repeated for every dose combination.

### 2.3. Rigidity

Because of safety considerations, most phase I trials will approach the estimated *MTC*_*θ*_ from below, using either constrained escalation [2,17] or start-up rules [4,18]. In the case of the PIPE design, toxicity results from the current cohort do not influence the estimated risk of DLT at other dose combinations because the risks are independent. At the beginning of a trial, if no toxicities are observed below the estimated *MTC*_*θ*_, then the contour remains unchanged. Only by observing either no toxicity above the estimated *MTC*_*θ*_ or a toxicity below the estimated *MTC*_*θ*_ can the contour change.

To explain this, consider a new study with *θ* = 0.3 and a weak prior, and let C^*(0)^ equal the most likely *MTC*_*θ*_ at the beginning of the trial. Suppose that the first cohort of two patients were administered a closest dose combination *d*_*kl*_ below C^*(0)^ and that a toxicity was observed. Then consider the monotonic contour *C*_2_ that is the same as C^*(0)^ apart from dose combination *d*_*kl*_, which lies above *C*_2_. Given the data and the weak prior, the posterior median probability *p*_*kl*_(*θ*|*Y*^(*m*)^) is less than 0.5. The ratio 
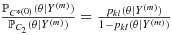
 is therefore less than 1, meaning that contour *C*_2_ is more likely than C^*(0)^ to be the new *MTC*_*θ*_, and hence, the contour changes. If no toxicities were observed in the first cohort then *p*_*kl*_(*θ*|*Y*^(*m*)^) is still greater than 1 − *p*_*kl*_(*θ*|*Y*^(*m*)^), and hence, C^*(0)^ remains the most likely *MTC*_*θ*_.

A consequence of this is that experimentation must take place on either side of the contour in order to allow it to move and hence avoid rigidity. Such issues have been discussed by Cheung [19] in the context of nonparametric dose-escalation designs. Varied experimentation using the sample size adaptive allocation to choose the next dose is an attractive design feature that can overcome problems of rigidity and allows trialists to look at many potentially promising dose combinations. A weighted randomisation strategy similar to that proposed in Equation (4) has also been used by Bekele and Shen [20] who found that without it their dose-finding procedure could ‘stick’ at lower doses.

### 2.4. Dose-skipping and safety constraints

It is often the case that a phase I clinical trial is escalated from the lowest possible dose combination, *d*_11_, and that dose skipping through the pre-defined levels of drugs A and B is prohibited. Such constraints have been described previously [2,17] and can easily be accommodated within the PIPE design.

#### 2.4.1. Neighbourhood constraint

**Figure 3:**
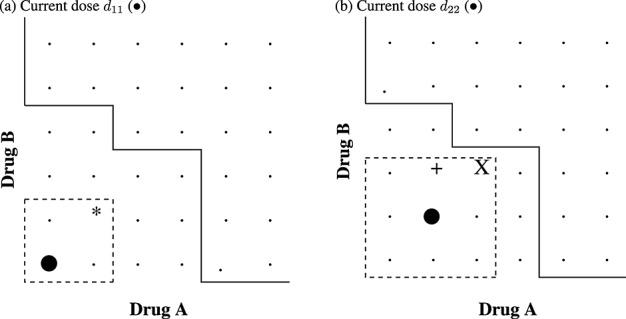
The set of admissible doses that are *closest and adjacent* (X), *adjacent but not closest* (+) and *largest* (*) to C^*(*m*)^ under a neighbourhood constraint. The solid line shows C^*(*m*)^. The dashed line shows the current neighbourhood constraint (i.e. only dose combinations within the dashed box are admissible).

A neighbourhood constraint forces the admissible doses for the next cohort to come from a restricted set of doses that are a maximum of one dose level higher or lower than the current experimented dose, both for drugs A and B. The closest and adjacent doses to the *MTC*_*θ*_ that can be identified given the current constraint, and as an example, are shown in Figure 3 for a trial that has its current cohort dosed at either (a) *d*_11_ or (b) *d*_22_. In example (a), the dashed box indicates the admissible doses under the neighbourhood constraint; there is one dose combination that is *largest*, *d*_22_, and there are no admissible dose combinations that are adjacent to the estimated *MTC*_*θ*_. Therefore, in this case, under both the closest and adjacent admissible dose strategies, we revert to selecting the *largest* dose combination to be the next administered dose. This has the effect of imparting *diagonal escalation* [17] in the first few cohorts of the trial until the bounding constraint reaches the estimated *MTC*_*θ*_. This occurs in example (b), where there is now one dose combination that is closest, *d*_33_, and two that are adjacent, *d*_23_ and *d*_33_, that could be chosen under the adjacent strategy.

#### 2.4.2. Non-neighbourhood constraint

In the previous section, the dose combination administered to the current cohort determines the local neighbourhood of admissible doses. In this section, a less constricted set of admissible doses is considered. Here, all previous doses administered are considered, and to avoid dose skipping, the constraint allows any dose that is a single-dose level higher, in either or both of drugs A and B, than *any previously* administered dose combination. This will allow greater exploration of all dose combinations.

**Figure 4:**
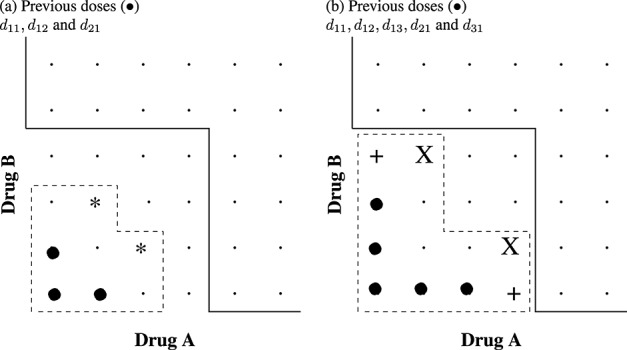
The set of admissible doses that are *closest and adjacent* (X), *adjacent but not closest* (+) and *largest* (*) to C^*(*m*)^ under non-neighbourhood dose-skipping constraint. The solid line shows C^*(*m*)^. The dashed line shows the current dose-skipping constraint (i.e. only dose combinations within the dashed box are admissible).

Similarly to the neighbourhood constraint, the closest and adjacent doses to the *MTC*_*θ*_ that are identified given the current constraint, and as an example, are shown in Figure 4 for two hypothetical trials. The previously administered drugs for the hypothetical trials are either (a) *d*_11_,*d*_12_ and *d*_21_ or (b) *d*_11_,*d*_12_,*d*_13_,*d*_21_ and *d*_31_ and are shown by the black circles in Figure 4. In example (a), the dashed box indicates the admissible doses under the non-neighbourhood constraint; there are two dose combinations that are *largest* and no admissible dose combinations that are adjacent to the estimated *MTC*_*θ*_. Therefore, in this case, under both the closest and adjacent admissible dose strategies, we revert to selecting one of the *largest* doses to be the next dose combination. In example (b), there are two dose combinations that are closest, *d*_24_ and *d*_42_, and four that are adjacent, *d*_24_, *d*_42_, *d*_14_ and *d*_41_, that could be chosen under the adjacent strategy.

#### 2.4.3. Safety constraint

Additionally, a safety constraint can be imposed to avoid any potential overdosing. Consider the expected probability of dose combination *d*_*ij*_ being above the *MTC*_*θ*_, averaged over the distribution of the monotonic contours. Let this probability be denoted as 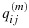 after *m* cohorts, which is written as


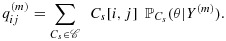


The safety constraint ensures that dose combination *d*_*ij*_ is excluded from the admissible dose set if 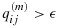, where *ε* is a prechosen constant. In the simulation studies that follow, we have found that choosing *ε* = 0.8 gives good operating characteristics. Averaging over the posterior distribution of the monotonic contours borrows strength across all the data currently collected inducing a dependence between the *q*_*ij*_ even though the *p*_*ij*_ are independent. The trial is terminated if there are no available dose combinations that satisfy the safety constraint.

### 2.5. The recommended phase II doses

At the end of the trial, we may wish to recommend more than one dose combination to take forward for further experimentation at phase II. To do this, after the last cohort *M* has been recruited, C^*(*M*)^ is estimated. Dose combinations that are *closest from below* to C^*(*M*)^ and have been experimented on during the trial are selected as the recommended phase II (RPII) doses. These two rules are chosen for safety reasons, because closest doses above C^*(*M*)^ may still be unbounded in their upper estimated toxicity risk if a safety constraint is not imposed.

## 3. Simulations

### 3.1. Simulation study 1

We investigate the operating characteristics for various PIPE designs and compare them to a popular six-parameter dual-agent dose-toxicity model [1]. For the six-parameter model, the dose combination for the next cohort is chosen based on the posterior mean toxicity risk closest to the TTL. The operating characteristics of this model have previously been assessed under a diagonal escalation constraint using four simulation scenarios [17], each drug having six dose levels. The true toxicity probabilities for the scenarios are displayed in Table I and are labelled as follows: scenario 1, in agreement with prior; scenario 2, toxic; scenario 3, asymmetric toxic; and scenario 4, flat. The TTL is set to be 30*%*; the dose combinations that have this TTL are shown in bold in Table I. Priors for the six-parameter model were assigned as stated in [1]. All other design settings for this model are explained in [17].

**Table I:** True toxicity probabilities for the four scenarios in simulation 1, with maximum tolerated doses shown in bold (doses within 0.025 of the target toxicity level).

	Drug A					
	
Drug B	0.2	0.5	0.7	0.8	0.9	0.95
	Scenario 1: in agreement with prior					
0.95	0.23	0.27	**0.30**	0.36	0.44	0.49
0.90	0.18	0.21	0.26	**0.30**	0.39	0.44
0.80	0.11	0.14	0.18	0.23	**0.30**	0.36
0.70	0.06	0.09	0.14	0.18	0.26	**0.30**
0.5	0.03	0.05	0.09	0.13	0.21	0.27
0.2	0.02	0.03	0.06	0.10	0.18	0.23
	Scenario 2: toxic					
0.95	0.23	**0.30**	0.45	0.50	0.55	0.60
0.9	0.18	0.21	**0.30**	0.45	0.50	0.55
0.8	0.11	0.14	0.18	**0.30**	0.45	0.50
0.7	0.06	0.09	0.14	0.18	**0.30**	0.45
0.5	0.03	0.05	0.09	0.13	0.21	**0.30**
0.2	0.02	0.03	0.06	0.10	0.18	0.23
	Scenario 3: asymmetric toxic					
0.95	**0.30**	0.38	0.48	0.58	0.68	0.78
0.9	0.22	**0.30**	0.40	0.50	0.60	0.70
0.8	0.17	0.25	0.35	0.45	0.50	0.60
0.7	0.12	0.20	**0.30**	0.40	0.45	0.55
0.5	0.06	0.14	0.24	0.34	0.39	0.49
0.2	0.02	0.10	0.20	**0.30**	0.35	0.45
	Scenario 4: flat					
0.95	0.265	**0.295**	0.325	0.355	0.385	0.415
0.9	0.250	**0.280**	**0.310**	0.340	0.370	0.400
0.8	0.235	0.265	**0.295**	0.325	0.355	0.385
0.7	0.220	0.250	**0.280**	**0.310**	0.340	0.370
0.5	0.205	0.235	0.265	**0.295**	0.325	0.355
0.2	0.190	0.220	0.250	**0.280**	**0.310**	0.340

In the PIPE design, the prior median probabilities of DLT are set to the true probabilities shown in scenario 1 (Table I), using either a strong or weak prior (Section 2.2). The priors chosen for the six-parameter model are as described in [17] and correspond to prior mean risks as in scenario 1.

One thousand simulated trials are used to investigate the operating characteristics; trials have a cohort size of 2, a sample size of 40, and escalate using a neighbourhood constraint after starting at the lowest dose combination, *d*_11_. The PIPE designs use a safety constraint with *ε* = 0.8. We compare different admissible dose sets (closest and adjacent) and the use of weighted randomisation or not. For comparison of computation efficiency, the PIPE design took 5.5 min to run 1000 simulations compared with 4.2 h for the six-parameter model.

### 3.2. Simulation study 2

To further investigate the performance of PIPE, the model is compared with the results from Braun and Jia [5] under seven further scenarios, labelled A–G that are reproduced in Table II. The prior distributions for PIPE are set to be the true DLT probabilities taken from scenario A. Each trial targets a toxicity level of 20%, with a total sample size of 50 and 1 patient per cohort. The starting dose is always the lowest dose, *d*_11_. Two thousand simulated trials are used to investigate the operating characteristics of PIPE versus (i) the generalised CRM (gCRM) [5], (ii) the latent contingency table approach of Yin and Yuan [3], and (iii) the copula model of Yin and Yuan [2]. The gCRM [5] is similar in spirit to earlier work for dose finding with two ordered groups [21,22] and can be seen as an extension of the single-agent logistic CRM allowing a separate intercept parameter for each level of the second drug. The three parametric models investigated use rescaled doses and priors as specified in [5]. All methods use a neighbourhood constraint. The PIPE method uses the closest doses chosen from the admissible dose set, and then further selected by the smallest sample size, a weak prior distribution (1/16) is specified, and a safety constraint with *ε* = 0.8 is used. The PIPE design took 2 min to run 2000 simulations.

**Table II:** True dose-limiting toxicity percentages for the seven scenarios in simulation 2 and as examined in [5].

		Drug A						Drug A			
Scenario	Drug B	1	2	3	4	Scenario	Drug B	1	2	3	4
A	1	4	8	12	16	E	1	8	18	28	29
	2	10	14	18	22		2	9	19	29	30
	3	16	20	24	28		3	10	20	30	31
	4	22	26	30	34		4	11	21	31	41

		Drug A						Drug A			
Scenario	Drug B	1	2	3	4	Scenario	Drug B	1	2	3	4
B	1	2	4	6	8	F	1	12	13	14	15
	2	5	7	9	11		2	16	18	20	22
	3	8	10	12	14		3	44	45	46	47
	4	11	13	15	17		4	50	52	54	55

		Drug A						Drug A			
Scenario	Drug B	1	2	3	4	Scenario	Drug B	1	2	3	4
C	1	10	20	30	40	G	1	1	2	3	4
	2	25	35	45	55		2	4	10	15	20
	3	40	50	60	70		3	6	15	30	45
	4	55	65	75	85		4	10	30	50	80

		Drug A									
Scenario	Drug B	1	2	3	4						
D	1	44	48	52	56						
	2	50	54	58	62						
	3	56	60	64	68						
	4	62	66	70	74						

### 3.3. Illustration of product of independent beta probabilities escalation to a single trial

**Figure 5:**
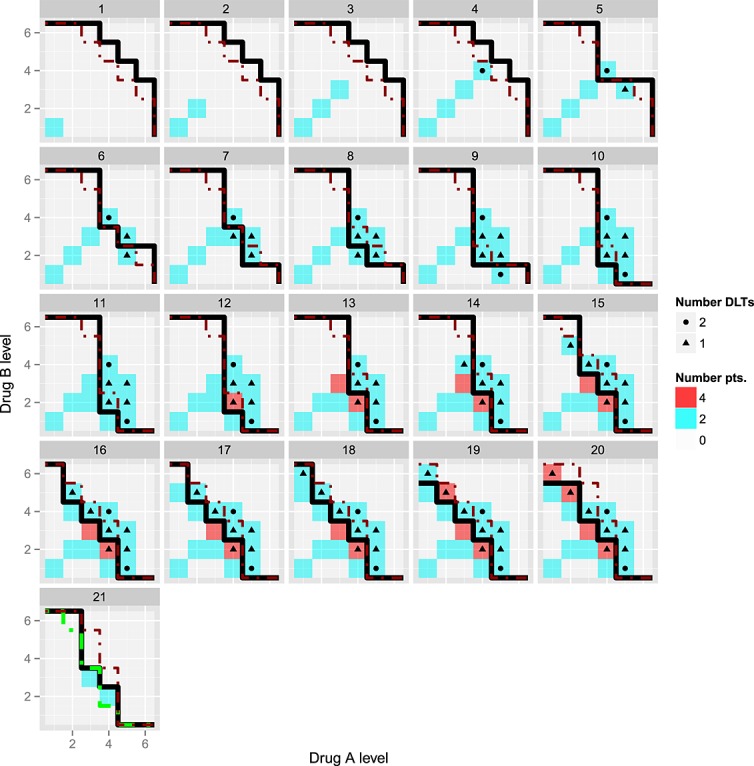
A single trial realisation simulated under scenario 3 using a neighbourhood constraint. Two patients per cohort were recruited sequentially from cohort 1 (top left panel) to cohort 20 (bottom right panel). Each subfigure *m* = 1,…,20 shows C^*(*m*)^ (black solid line), the upper toxicity constraint (dashed red contour) based on doses whose posterior probability 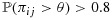 as well as current and past experimentation (shaded boxes) and dose-limiting toxicities (symbols). Cohort 21 (bottom left panel) additionally shows the recommended phase II doses at the end of the trial (blue boxes) as well as the true *MTC*_*θ*_ (dashed green line).

To illustrate the PIPE design working in practice, results from a single simulated trial are presented. Escalation uses a neighbourhood constraint, with admissible doses chosen from those closest to the estimated *MTC*_*θ*_ and with the smallest sample size. The toxicity risk at each dose combination is simulated from scenario 3, where the true DLT risks are higher than expected under the prior. Figure 5 shows the escalation from cohort 1 (top left panel) to cohort 20 (bottom right panel). The black contour is the estimated *MTC*_*θ*_ before the current cohort's outcome is observed, whilst the red contour is the safety constraint; no dose combination above this contour can be selected for the next cohort. The coloured boxes and symbols show the accumulating data on experimentation and toxicity, respectively. Cohort 21 (bottom left panel) shows the estimated *MTC*_*θ*_ at the end of the trial, the true *MTC*_*θ*_ (dashed green contour) and the RPII doses (blue boxes).

It is clear from this figure that the *MTC*_*θ*_ contour adapts only when a DLT is observed below the contour or no DLT is observed above the contour. Two out of two DLTs are observed in cohort 4, causing the safety constraint to exclude this and any higher dose levels from experimentation in cohort 5. In this example, the trial adapts well to the higher than expected DLT rate, with 11/40 DLTs observed during the trial and with two RPII doses, both adjacent to the true *MTC*_*θ*_.

### 3.4. Results of simulation study 1

#### 3.4.1. Experimentation

In Table III, we report the percentage of experimentation against the true toxicity probability across all simulated trials and for each of the four scenarios and five designs. All results shown are for a neighbourhood escalation constraint with weak priors used for the PIPE designs. Given that the TTL is 0.3, the ideal experimentation would predominantly be in the 25–34% toxicity range of the table, with some experimentation at safer doses as the initial dose combination is *d*_11_. Note that for scenario 4, there are no dose combinations that have toxicity probabilities in ranges 0–14 or 46+, and hence, experimentation is zero for these cells in the table.

**Table III:** Experimentation and recommendation percentages for the six-parameter model and product of independent beta probabilities escalation designs using neighbourhood escalation constraints for simulation study 1.

	Experimentation toxicity (%)					Recommendation toxicity (%)					Mean number recommended doses
		
Design	0–14	15–24	25–34	35–45	46+	0–14	15–24	25–34	35–45	46+	
Scenario 1: in agreement with prior											
Six-parameter model	17	17	42	24	0	1	17	59	23	0	0.7
PIPE: *Closest*, Min. *S*	20	24	44	12	0	3	28	56	13	0	2.7
PIPE: *Adjacent*, Min. *S*	26	35	33	6	0	3	30	53	14	0	2.3
PIPE: *Closest*, WR *S*	20	24	44	12	0	3	29	56	12	0	2.7
PIPE: *Adjacent*, WR *S*	26	35	33	6	0	3	31	52	15	0	2.3
Scenario 2: toxic											
Six-parameter model	18	17	37	21	8	1	20	56	22	2	1.0
PIPE: *Closest*, Min. *S*	21	24	32	19	4	4	34	45	16	3	3.0
PIPE: *Adjacent*, Min. *S*	28	34	25	11	3	4	34	40	19	3	2.5
PIPE: *Closest*, WR *S*	21	24	34	18	3	4	34	45	16	1	2.8
PIPE: *Adjacent*, WR *S*	28	34	24	11	3	4	37	37	18	4	2.3
Scenario 3: asymmetric toxic											
Six-parameter model	12	9	28	42	10	1	11	35	50	4	0.4
PIPE: *Closest*, Min. *S*	13	13	29	36	9	2	14	35	43	6	2.5
PIPE: *Adjacent*, Min. *S*	16	18	28	32	6	2	14	35	42	8	2.1
PIPE: *Closest*, WR *S*	13	12	30	36	9	1	15	36	41	7	2.4
PIPE: *Adjacent*, WR *S*	15	18	27	33	6	2	14	33	42	9	1.9
Scenario 4: flat											
Six-parameter model	0	29	59	13	0	0	12	75	12	0	0.6
PIPE: *Closest*, Min. *S*	0	25	63	12	0	0	7	76	17	0	2.3
PIPE: *Adjacent*, Min. *S*	0	22	70	9	0	0	4	78	18	0	2.0
PIPE: *Closest*, WR *S*	0	24	64	12	0	0	6	77	16	0	2.2
PIPE: *Adjacent*, WR *S*	0	21	70	9	0	0	4	75	20	0	1.9

The PIPE designs used the *weak prior*, a safety constraint (Section 2.4.3) and dose allocation by inverse sample size weighted randomisation (WR *S*) or by sample size alone (Min. *S*).

From the results in Table III, there are small differences between PIPE designs using the weighted randomisation and PIPE designs using the minimum sample size. There are marked differences between the operating characteristics of designs using adjacent or closest admissible doses. Overall, the adjacent PIPE designs tend to be more conservative than the closest designs with experimentation more often at less toxic dose combinations. Therefore, for comparison with the six-parameter model, we focus on the closest PIPE designs. These designs have similar experimentation with the six-parameter models at the target dose combinations with the closest PIPE designs having less overdosing in all the scenarios than the model-based design.

The simulations were repeated to compare the performance with the non-neighbourhood constraint. For the non-neighbourhood designs, experimentation was slightly more varied than the neighbourhood constraint, but the recommended doses did not qualitatively change.

#### 3.4.2. Recommendation

The mean number of recommended doses and the percentage of RPII dose combinations for each true toxicity range are given in Table III. Again, the main differences amongst the PIPE designs were between closest and adjacent admissible dose constraints rather than between weighted randomisation or not. The adjacent PIPE designs recommended the most toxic dose combinations slightly more often than the closest PIPE designs with the closest PIPE designs being better at recommending dose combinations close to the TTL. In comparing the closest PIPE designs with the six-parameter model-based design, the RPII doses from the closest PIPE designs were slightly less likely to be in target toxicity range 25–34% for scenario 1, slightly more likely in scenarios 3 and 4 and much less likely in scenario 2, but were also less likely to be in the toxic range 35+% except for scenario 4. On average, the closest PIPE designs recommended over three times the number of dose combinations than the six-parameter model-based designs. This is a direct result of the more varied experimentation.

#### 3.4.3. Choice of prior strength

We repeated all the aforementioned simulations using the strong prior, results of which are shown in the Supporting information (Supporting information Table 1). For scenario 1 with a strong prior, there was around 4% to 10% more experimentation at the targeted (25–34%) toxicity range, compared with a PIPE design using a weak prior. For scenario 2, the overdosing (35+%) was slightly higher ≈2*%*; for scenarios 3 and 4, the overdosing was over 10% higher with most of the closest designs. It is clear that even with a prior sample size of 1 per dose combination, the benefits gained when the prior estimate is correct are small compared with the substantial overdosing seen when the prior estimates are incorrect. The recommended dose combinations had a similar pattern of selecting more toxic dose combinations and recommending on average similar numbers of dose combinations.

### 3.5. Results of simulation study 2

The operating characteristics of the PIPE method and the three parametric models from [5] are shown in Table IV. Results from the three parametric models are reproduced from Table 2 in [5]. The experimentation and recommendation percentages are reported against the true toxicity probabilities across all simulated trials for each of the seven scenarios and four different designs. Note that the simulations from [5] were set up to consider single MTDs and emphasise dosing at the target. However, because PIPE is estimating the whole toxicity contour, there are dose combinations that are not exactly at the TTL (20%) but can be recommended as they are just below the toxicity contour. On the whole, we therefore emphasise dosing and recommendations within 10% of the TTL.

**Table IV:** Experimentation and recommendation percentages for the generalised continual reassessment method [5], Yin and Yuan (2009) [3] (denoted YY09a), Yin and Yuan (2009) [2] (denoted YY09b) and product of independent beta probabilities escalation models for simulation study 2.

		Recommendation percentages a				Experimentation percentages			
			
Scenario	Model	At *θ* of *θ*	Within 1–10% of *θ*	>10%	None	At *θ*	Within 1–10% of *θ*	>10% of *θ*	None
A	gCRM	10	82	3	5	6	72	17	5
	YY09a	13	82	5	0	13	72	15	0
	YY09b	11	81	6	2	10	70	20	0
	PIPE	10	88	3	0	8	87	5	0
B	gCRM	0	94	3	3	0	87	13	0
	YY09a	0	99	1	0	0	86	14	0
	YY09b	0	96	4	0	0	71	29	0
	PIPE	0	83	17	0	0	82	18	0
C	gCRM	45	39	5	11	30	41	18	11
	YY09a	41	50	5	4	27	54	16	3
	YY09b	42	47	5	6	29	55	11	5
	PIPE	29	59	7	5	19	46	34	2
D	gCRM	0	0	4	96	0	0	22	78
	YY09a	0	0	1	99	0	0	20	80
	YY09b	0	0	1	99	0	0	16	84
	PIPE	0	0	1	99	0	0	37	63
E	gCRM	9	70	14	7	5	56	32	7
	YY09a	6	65	27	2	9	55	34	2
	YY09b	7	67	25	1	6	54	38	2
	PIPE	11	84	4	1	9	77	13	1
F	gCRM	13	70	6	11	10	64	16	10
	YY09a	14	76	6	4	7	75	14	4
	YY09b	12	74	7	7	7	77	9	7
	PIPE	12	75	11	2	12	69	18	2
G	gCRM	25	68	5	2	18	57	24	1
	YY09a	12	76	12	0	3	71	26	0
	YY09b	15	72	13	0	7	61	32	0
	PIPE	9	62	29	0	14	54	31	0

Results from the three parametric models are reproduced from Table 2 in [5]. The PIPE design used a weak prior, a safety constraint with *ε* = 0.8 and dose allocation by minimum sample size.

gCRM, generalised continual reassessment method; PIPE, product of independent beta probabilities escalation.

a As PIPE can choose more than one recommended phase II dose, the statistics for PIPE give the percentage of times each dose is selected per selected dose. A decision to recommend no phase II dose is made if the trial is stopped early for safety.

#### 3.5.1. Experimentation

There is considerable variation in performance of the methods across the scenarios. For scenarios A and E, PIPE has 15% more experimentation on dose combinations within 10% of the target than all the other methods. For scenario B, the results are similar between methods (although YY09b does less well). In scenario F, PIPE has 81% experimentation on dose combinations within 10% of the target; this is slightly less than YY09a and YY09b but better than gCRM. In scenario G, PIPE has 31% of dose combination experimentation at dose combinations over 10% of the TTL, which is similar to YY09b but up to 7% worse than the other methods. In the more unsafe scenarios C and D, PIPE has at least 16% more experimentation on dose combinations that are above 10% of the target than the other methods. However, this excess in overdosing can be rectified by taking a lower safety threshold of 60% rather than 80*%* (results not shown).

#### 3.5.2. Recommendation

The dose combinations that are recommended at the end of the trial are more similar amongst the methods than the experimentation percentages. PIPE is less likely to have no RPII doses than the other methods. In scenario A, PIPE is the best performing method with 98*%* dose recommendations coming within 10*%* of the target. In scenario B, PIPE performs worse and takes a more conservative approach by recommending doses below the target 17% of the time. This phenomenon arises because of the single patient cohort size, where the safety constraint often cannot adapt quickly after a single DLT is observed. We would generally recommend a larger cohort size when using PIPE (a cohort size of 3 reduced the below target recommendation percentage to 12*%*, results not shown). The results for scenarios C, D and F give very similar recommendations amongst the methods, although in scenario C, given the multiple recommendations of the PIPE design, more dose combinations are recommended within 1–10% of the TTL rather than exactly at the target. In scenario E, PIPE outperforms all the other methods by at least 15*%* in terms of recommendations within 10*%* of the TTL. One possible reason for this is the asymmetric *MTC* in this scenario, which parametric-based methods may fail to capture adequately. PIPE does worse in scenario G and, similar to scenario B, is conservative in dose combination recommendations.

## 4. Discussion

We have introduced a simple design for dual-agent dose escalation based on the product of independent beta probabilities and estimation of monotonic contours. The PIPE design does not rely on any underlying parametric model and relies only on the monotonicity assumption. The philosophy of the design lies in selecting from a number of competing doses around the estimated MTC rather than fixing on a single-dose combination. Nevertheless, we have shown that PIPE has comparable operating characteristics with the six-parameter dose-escalation method from [17], whilst still retaining the advantage of being a nonparametric, fast and interpretable approach. Furthermore, PIPE generally compares favourably with other parametric dose-escalation methods, as shown in our second simulation study. In some scenarios, PIPE outperforms the parametric methods, for example, when the prior probabilities are correct (scenario A) and when the parametric models can not fully describe the complexity of the data such as in scenario E, where the toxicity surface is asymmetric. However, we acknowledge that in scenarios where nearly all drug combinations were more toxic than expected (scenarios C and D), the varied experimentation properties of PIPE along the estimated contour resulted in a higher percentage of experimentation at less safe dose combinations. This is the trade-off associated with using a design with few underlying assumptions, although the operating characteristics of PIPE in these scenarios can be improved further by using a stricter safety constraint.

The disadvantage of using a nonparametric design is that there is less borrowing of information across the dose space, and hence, researchers should be aware that rigidity can cause these designs to behave suboptimally. In the context of the TPI design [13,14] for single-agent trials, this shortcoming was deemed acceptable because of the benefit of very simple decision rules. In the dual-agent setting, the decision rules are naturally more complex. With PIPE, we have carefully considered the conditions under which model rigidity can occur and propose a design that decreases the chances of this by promoting varied experimentation.

We have shown only two possible ways of selecting between the admissible doses, either by selecting the combination with the lowest sample size or using the inverse of the sample size in a weighted randomisation. It is possible to consider other dose selection algorithms such as selecting a dose combination that is most informative for estimation of the MTC; this may be a more optimal approach to estimating the MTC accurately, although our simple approach based on sample size effectively allows varied experimentation. We also considered using the value of the probability distribution function at the TTL for each admissible dose (choosing the dose with the highest value) but found that this could induce rigidity in the design. As an example, suppose that only two dose combinations are admissible throughout: the current dose combination in which we have observed one DLT out of two patients and an unexperimented dose. Both dose combinations have *Beta*(0.39,0.61) priors, corresponding to a prior median DLT rate of 0.3 from a sample size of 1. Then even if no more DLTs are seen, we would continue to recommend the experimented dose even after recruiting 10 patients to that dose, that is, the PDF evaluated at *θ* = 0.3 is higher for the experimented dose even after 10 patients: *f*(*θ*,0.39 + 1,0.61 + 9) > *f*(*θ*,0.39,0.61).

The nonparametric monotonic contours approach we have suggested for selecting dose combinations can also be used at the end of the trial to estimate the whole dose-toxicity surface. By considering different values of *θ* = 0.1,0.2,…,0.9 and selecting the resulting most likely monotonic contours, it is possible to construct the most likely dose-toxicity surface.

Throughout this paper, we have not specified whether the two agents are ‘novel’ or established drugs. If the agents are established, then the marginal priors could be taken directly from a systematic review and predictive toxicity risks, and effective sample size can be incorporated directly into the priors; the prior strength is therefore fixed by this external evidence. We also do not need to start dose escalations from *d*_11_ but could start at any dose level that is believed to be safe.

An important aspect of the PIPE design is that the priors are based on elicited or historical information; that is, the priors directly reflect the estimated risk of toxicity at each dose combination and its uncertainty. For other published methodologies, sometimes priors are chosen via simulation to give the desired operating characteristics of the design [12]; the final prior is therefore a design prior and may not bear any resemblance of the clinicians prior belief of the toxicity surface. The flexibility of the priors used in the PIPE design means that the method can be easily and fairly compared with other parametric model-based designs because we can match the median and variance of the prior toxicity probabilities at each dose combination to those implied by the parametric model. However, the distributions of the imposed priors from any parametric model may not be beta (and of course will be correlated between doses), and hence, any comparison is only approximate. This difficulty was shown in [17] when it was not possible to form comparable priors for a copula-type model and the six-parameter model.

The other major advantage of the PIPE design is the ability to identify multiple RPII doses for future study due to allowing more varied experimentation. Many existing methods focus on recommending a single-dose combination, which may limit future exploration.

We have shown that a PIPE design based on selecting doses closest to the estimated MTC is generally preferable over selecting doses that are only adjacent. The operating characteristics seen in simulation study 1 show better experimentation and recommendation percentages using the closest design. The adjacent approach should therefore be reserved only for designs where large varied experimentation around the MTC is desirable and in particular where the dose-toxicity surface is relatively smooth to permit experimentation further from the MTC (e.g. as in scenario 4).

A PIPE-based design could feasibly be applied to a single-agent trial by setting drug B to have a single-dose level. In this case, the MTC would just be a point that bisects the dose range of drug A, and there would always be two doses that are closest to the MTC, one below and one above the bisection point. However, the operating characteristics of such an approach need more careful consideration, and in this situation, it may not always be desirable to alternate between two admissible doses.

In summary, the PIPE method provides a simple and intuitive design for dual-agent trials, and we have shown that it performs well in a selection of scenarios. It bears similarities with the TPI approach [13,14]. We believe PIPE is easier to implement as it does not require specification of tuning parameters, although a modified version of the TPI approach has been proposed to allow the algorithm to be more automated [14]. Computationally, the PIPE method is fast, and R, Stata and Excel code are available from the authors on request.

## Supporting information

Supporting info item
