# Supplementary material for: A product of independent beta probabilities dose escalation design for dual-agent phase I trials
Source: Stat Med. 2015 Jan 29;34(8):1261–76. doi: 10.1002/sim.6434 (PMC4409822; doi:10.1002/sim.6434)
Supplement: Supplementary file 1 — Supporting info item [file sim0034-1261-sd1.pdf]

Supporting Information

A. Proof: Number of possible monotonic contours

The following result can be proved by induction,

$$\sum_{k=0}^{n} \left(\begin{array}{c} r+k \\ k \end{array} \right) = \left(\begin{array}{c} r+n+1 \\ n \end{array} \right). \quad (5)$$

Take the situation where there are I levels of drug A and J levels of drug B. Each contour is represented by an I by J matrix of 1s and 0s that partitions the dose combinations into being above the MTC or not, we call this the binary matrix, P.

We are going to assume the partitioning matrix must be monotonic in rows and columns *i.e.* $P[i,j] \geq P[i-1,j]$ and $P[i,j] \geq P[i,j-1]$. Note under monotonicity there can only be $J+1$ possible rows, shown below,

$$(1,1,\ldots,1),(0,1,\ldots,1),...,(0,\ldots,0,0),$$

label these row vectors as $r_1,\ldots,r_{J+1}$.

Taking only monotonic partitioning matrices, let the number of such matrices having the row r_j as the last (Ith) row be n_{Ij}. By definition, when $I=1$ all the $n_{1j}=1$.

So it follows that the total number of monotonic matrices, T_{IJ}, is the summation of these counts,

$$T_{IJ} = \sum_{j=1}^{J+1} n_{Ij}.$$

We will use proof by induction to prove that the formula for n_{ij} can be written as,

$$n_{ij} = \frac{(i+j-2)!}{(i-1)!(j-1)!} = \left(\begin{array}{c} i+j-2 \\ j-1 \end{array} \right), \quad (6)$$

This formula holds for the $i=1$ case. Now consider the case of having a dose grid of dimensions l by J and then add an extra dose level, *i.e.* row, to make a $l+1$ by J dose combination grid. It is easy to show that

- we can only add row r_1 to the n_{l1} matrices with the last row of r_1,
- we can add row r_2 to the n_{l1} matrices ending in r_1 and the n_{l2} matrices ending in r_2, and so on until
- we can add row r_{J+1} to all $(\sum n_{li})$ matrices

It follows simply that the n_{ij}s are defined recursively

$$n_{ij} = \sum_{k=1}^{j} n_{(i-1)k}.$$

 Copyright © 2010 John Wiley & Sons, Ltd.

Using equation 6 we can plug in the values of $n_{(i-1)k}$ and thus prove that the formula is correct.

$$\begin{aligned} n_{ij} &= \sum_{k=1}^{j} n_{(i-1)k} \\ &= \sum_{k=1}^{j} \binom{i-1+k-2}{k-1} \\ \text{By transformation} &= \sum_{k=0}^{j-1} \binom{i-1+k-1}{k} \\ \text{By using equation 5} &= \binom{i-2+j-1+1}{j-1} \\ &= \binom{i+j-2}{j-1} \end{aligned}$$

Given this definition of n_{ij} we can then calculate the total number of monotonic contours by the following proof.

$$\begin{aligned} \sum_{j=1}^{J+1} n_{Ij} &= \sum_{j=1}^{J+1} \frac{(I+j-2)!}{(I-1)!(j-1)!} \\ &= \sum_{j=1}^{J+1} \binom{I-1+j-1}{j-1} \\ &= \sum_{k=0}^{J} \binom{I-1+k}{k} \\ &= \binom{I-1+J+1}{J} = \frac{(I+J)!}{I!J!} \end{aligned} \quad (7)$$

B. Supporting information

B.1. Results from PIPE designs using a strong prior for simulation study 1

Supporting Information Table 1 shows the experimentation and recommendation percentages, respectively, of the PIPE design using strong priors and comparing the results with the six-parameter model design.

Design	Experimentation Toxicity (%)					Recommendation Toxicity (%)					Mean number
	0-14	15-24	25-34	35-45	46+	0-14	15-24	25-34	35-45	46+	recommended doses
Scenario 1: in agreement with prior											
Six-parameter model	17	17	42	24	0	1	17	59	23	0	0.7
PIPE: *Closest*, Min. S	16	21	52	11	0	1	28	61	10	0	2.9
PIPE: *Adjacent*, Min. S	23	37	37	4	0	2	29	59	9	0	2.5
PIPE: *Closest*, WR S	16	20	54	9	0	1	25	66	8	0	2.6
PIPE: *Adjacent*, WR S	22	38	37	3	0	1	30	62	7	0	1.9
Scenario 2: toxic											
Six-parameter model	18	17	37	21	8	1	20	56	22	2	1.0
PIPE: *Closest*, Min. S	16	21	39	21	3	2	33	49	15	1	3.2
PIPE: *Adjacent*, Min. S	25	35	27	12	1	2	34	42	19	2	2.5
PIPE: *Closest*, WR S	16	19	42	22	2	1	32	51	15	1	2.6
PIPE: *Adjacent*, WR S	25	34	29	11	1	2	35	42	20	1	1.9
Scenario 3: asymmetric toxic											
Six-parameter model	12	9	28	42	10	1	11	35	50	4	0.4
PIPE: *Closest*, Min. S	10	5	24	47	15	0	9	32	51	8	2.7
PIPE: *Adjacent*, Min. S	12	14	28	39	8	0	9	33	48	9	2.1
PIPE: *Closest*, WR S	10	3	24	48	15	0	7	31	54	8	2.1
PIPE: *Adjacent*, WR S	12	13	29	39	6	1	10	34	48	7	1.6
Scenario 4: flat											
Six-parameter model	0	29	59	13	0	0	12	75	12	0	0.6
PIPE: *Closest*, Min. S	0	10	67	22	0	0	1	78	22	0	2.8
PIPE: *Adjacent*, Min. S	0	13	77	10	0	0	1	79	20	0	2.2
PIPE: *Closest*, WR S	0	10	69	20	0	0	1	77	22	0	2.3
PIPE: *Adjacent*, WR S	0	13	78	9	0	0	1	81	18	0	1.6

Supporting Information Table 1. Experimentation and recommendation percentages for the six-parameter model and PIPE designs using neighbourhood escalation constraints for simulation study 1. The PIPE designs used the **strong prior**, safety constraint (see Section 2.4.3) and dose allocation by inverse sample size weighted randomisation (WR S) or by sample size alone (Min. S)

 Copyright © 2010 John Wiley & Sons, Ltd.
